# Selective Enrichment of Chlorogenic Acid and Related Phenolic Acids from Spent Coffee Grounds by Ultrasound-Assisted Extraction with Deep Eutectic Solvents

**DOI:** 10.3390/foods15101743

**Published:** 2026-05-14

**Authors:** Chunqing Shi, Xiaoqing Li, Yulian Gong, Keqin Liao, Jiebao Long, Jie Xie, Yuxi Chen, Yitong Li, Bijian Zeng

**Affiliations:** 1 College of Biology and Food Engineering, Guangdong University of Education, 351 Xingangzhong Road, Haizhu District, Guangzhou 510303, China; 13686485026@163.com (C.S.); lixiaoqing318@163.com (X.L.); gongyulian@gdei.edu.cn (Y.G.); liao2442812006@163.com (K.L.); longjiebao99@163.com (J.L.); xiejie@gdei.edu.cn (J.X.); chenyuxi327@163.com (Y.C.); 18046907169@163.com (Y.L.); 2 School of Food Science and Engineering, South China University of Technology, 381 Wushan Road, Tianhe District, Guangzhou 510641, China

**Keywords:** SCGs, deep eutectic solvent, chlorogenic acid, ultrasound-assisted extraction, response surface methodology, UPLC-Q-TOF-MS

## Abstract

Spent coffee grounds (SCGs), a major by-product of coffee consumption, remain an underused source of chlorogenic acid (CGA) and other phenolic constituents. This study investigated an ultrasound-assisted extraction strategy using deep eutectic solvents (DESs) to improve the recovery and phenolic-acid enrichment of SCGs. Among the tested DES formulations, the betaine–acetic acid system gave the best CGA extraction performance and was therefore used for further optimization by response surface methodology. The optimized process, conducted at a liquid-to-solid ratio of 28 mL/g, 75 °C, and 50 min, produced a CGA yield of 15.18 mg CGA/g dried SCG powder, markedly exceeding that achieved with 70% ethanol under comparable conditions. Structural and chemical characterizations helped explain this improvement. Scanning electron microscopy revealed that the DES-based process caused more evident disruption of the SCG matrix, which favored solvent penetration and mass transfer. Fourier-transform infrared spectroscopy confirmed the formation of a hydrogen-bonding network between betaine and acetic acid. Ultra-performance liquid chromatography–quadrupole time-of-flight mass spectrometry further revealed that the betaine–acetic acid extract was mainly composed of CGA and hydroxycinnamic acid derivatives. The purified extract also displayed strong in vitro antioxidant capacity. Overall, the betaine–acetic acid DES combined with ultrasound provides an effective green approach for recovering CGA-rich phenolic extracts from SCGs.

## 1. Introduction

Large quantities of spent coffee grounds (SCGs) are generated after coffee brewing, and their disposal remains a practical challenge for both waste management and resource utilization. Rather than being treated only as a low-value residue, SCGs can serve as a renewable source of bioactive compounds, including chlorogenic acid (CGA), caffeine, fatty acids, and flavonoids [1]. CGA and its derivatives are among the major phenolic constituents in SCGs and mainly include mono- and dicaffeoylquinic acid derivatives, such as 3-caffeoylquinic acid, 4-caffeoylquinic acid, 5-caffeoylquinic acid, and related isomers [2]. Owing to their antioxidant, anti-inflammatory, antibacterial, and antiviral properties, these compounds have attracted increasing interest in food, pharmaceutical, and functional ingredient applications [3,4,5,6]. Therefore, developing an efficient extraction strategy for CGA and related phenolic acids is important for improving the value-added use of SCGs.

The recovery of CGA from plant-derived materials has traditionally relied on hot-water extraction or organic solvents such as methanol and ethanol [7,8]. Although these approaches are technically simple, they often involve high solvent consumption, limited selectivity, and environmental or safety concerns [9,10]. Physical intensification methods, including ultrasound-assisted extraction (UAE) and microwave-assisted extraction (MAE), have been introduced to accelerate mass transfer and shorten extraction time [11,12]. However, the extraction efficiency of these techniques still depends strongly on the compatibility between the solvent, the target compounds, and the sample matrix. For a complex lignocellulosic residue such as SCGs, an extraction system should ideally combine matrix disruption, efficient solubilization of phenolic acids, and reduced reliance on conventional organic solvents.

Deep eutectic solvents (DESs) provide a flexible solvent platform for this purpose. They are typically formed by combining a hydrogen bond acceptor (HBA) with a hydrogen bond donor (HBD), producing a liquid system whose physicochemical properties can be adjusted by changing the component type and molar ratio [13]. Compared with single-component organic solvents, DESs can create a solvent microenvironment rich in hydrogen-bonding interactions. This feature is particularly relevant for CGA, which contains hydroxyl, carboxyl, and ester groups capable of interacting with DES components. Previous studies have applied DESs to the extraction of various natural products, including isoflavones, polysaccharides, polyphenols, and phenolic acids [14,15,16]. DES-based systems have also been reported to improve CGA recovery from plant materials such as honeysuckle and sunflower residues [17,18,19]. Nevertheless, the use of DESs for selectively recovering CGA and related phenolic acids from SCGs remains insufficiently explored.

Combining ultrasound with DESs may further improve extraction performance through complementary effects. Ultrasound can loosen or damage the compact structure of plant and food-waste matrices through cavitation and mechanical effects, thereby facilitating solvent entry and solute diffusion. At the same time, DESs can promote the dissolution of phenolic acids by providing a suitable polarity-related environment and multiple hydrogen-bonding sites. In such a system, ultrasound mainly enhances access to intracellular or matrix-bound compounds, whereas the DES phase contributes to solubilization and stabilization of the released phenolic constituents. This combination is therefore suitable for extracting phenolic acids from SCGs, where both structural resistance of the matrix and solvent affinity for target compounds affect the final yield.

Current studies on DES-based valorization of SCGs remain limited, and many reported systems are based on choline chloride-containing DESs [20,21]. Exploring alternative HBAs may broaden the solvent choices available for SCG utilization. Betaine is a naturally derived zwitterionic compound with strong hydrogen-bonding potential and good compatibility with food-related applications. When combined with suitable HBDs, betaine-based DESs may provide a distinct solvent environment for the extraction of CGA and related phenolic acids. However, the performance of betaine-based DESs in ultrasound-assisted extraction from SCGs, as well as their influence on phenolic-acid enrichment and antioxidant activity, has not been fully clarified.

In this study, a series of choline chloride- and betaine-based DESs were screened for the ultrasound-assisted extraction of CGA from SCGs. After selecting the optimal DES, the extraction conditions were optimized using a Box–Behnken design within response surface methodology (RSM), with 70% ethanol included as a reference solvent. Fourier-transform infrared spectroscopy (FT-IR) was used to examine the hydrogen-bonding interactions involved in DES formation, and scanning electron microscopy (SEM) was applied to observe the morphological changes in SCGs after extraction. In addition, ultra-performance liquid chromatography–quadrupole time-of-flight mass spectrometry (UPLC-Q-TOF-MS) was used to characterize the phenolic-acid profile of the DES extract, and 1,1-diphenyl-2-picrylhydrazyl (DPPH), 2,2′-azino-bis(3-ethylbenzothiazoline-6-sulfonic acid) (ABTS), and ferric reducing antioxidant power (FRAP) assays were performed to evaluate its in vitro antioxidant activity. This work aims to provide a green extraction strategy for recovering CGA-rich phenolic extracts from SCGs and to clarify the role of betaine–acetic acid DES in phenolic acid enrichment.

## 2. Materials and Methods

### 2.1. Materials and Chemicals

SCGs were collected from Starbucks and identified as Arabica coffee residues. Before extraction, the samples were dried at 60 °C until a constant weight was reached, ground to powder, and sieved through a 40-mesh screen. Because the dried material was used directly for extraction, CGA yield was calculated without additional moisture correction. The prepared SCG powder was stored in sealed containers at 4 °C.

Betaine, choline chloride, acetic acid, ethylene glycol, sorbitol, propylene glycol, 1,4-butanediol, citric acid, and potassium bromide were obtained from Macklin Biochemical Technology (Macklin, Shanghai, China). ABTS, DPPH, and TPTZ were supplied by Suao Biotechnology (Suao, Guangzhou, China). Chlorogenic acid (HPLC ≥ 98%) was purchased from Shanghai Yuanye Bio-Technology (Yuanye, Shanghai, China). Macroporous resin was provided by Sanxing Resin (Sanxing Resin, Bengbu, China). Ethanol, methanol, acetonitrile, formic acid, ammonium formate, concentrated phosphoric acid, ferric chloride hexahydrate, ferrous sulfate heptahydrate, vitamin C, and phosphate-buffered saline (PBS) reagents were also used in this study. Unless otherwise specified, all reagents were of analytical grade.

#### 2.1.1. Preparation of Standard Solutions

A CGA stock solution was prepared in 20% (*v*/*v*) methanol and diluted to obtain calibration standards over the range of 0.003125–0.100 mg/mL. The prepared standards were used for HPLC quantification of CGA.

#### 2.1.2. Preparation of Reagents and Other Solutions

DPPH solution (0.2 mM), ABTS stock solution (7 mM), potassium persulfate solution (2.5 mM), FRAP working reagent, FeSO_4_·7H_2_O standards, and VC control solutions were freshly prepared as described in the corresponding antioxidant assay sections. Sample and VC solutions for the DPPH and ABTS assays were prepared in 50% (*v*/*v*) ethanol at 6.25–100 μg/mL, whereas those for the FRAP assay were prepared at 40–120 μg/mL.

### 2.2. Preparation of DESs

The DESs were prepared using a heating-assisted mixing procedure based on a previously reported method [22]. As listed in Table 1, betaine and choline chloride were used as hydrogen bond acceptors (HBAs), while sorbitol, acetic acid, 1,4-butanediol, citric acid, ethylene glycol, and propylene glycol served as hydrogen bond donors (HBDs). For each DES, the HBA and HBD were combined at a molar ratio of 1:2, followed by the addition of deionized water at 25% (*w*/*w*). The mixture was placed in a round-bottom flask and stirred at 50 °C until a clear and homogeneous liquid was formed. To describe the solvent characteristics of the tested systems, Table 1 also includes polarity-related solvent type information classified according to the HBD category and the expected hydrogen-bonding/polar features of the DES components.

### 2.3. Ultrasound-Assisted DES Extraction of CGA from SCGs

Each extraction was performed by adding 25 mL of the prepared DES to 1.0 g of SCG powder. The mixture was sonicated in a KQ-500E ultrasonic cleaning bath (Kunshan Ultrasonic Instrument Co., Ltd., Kunshan, China) for 40 min at 40 kHz and 70% power. The bath temperature was set to 60 °C and controlled by the built-in temperature-control system. After extraction, the suspension was centrifuged at 6000 rpm for 10 min. The supernatant was filtered through a 0.22 μm PTFE syringe filter and diluted 20-fold with 20% (*v*/*v*) methanol before HPLC analysis.

For comparison, 70% (*v*/*v*) ethanol was used as the reference extraction solvent. This reference extraction was conducted with the same liquid-to-solid ratio, ultrasonic frequency, power setting, extraction temperature, and extraction time as the DES extraction; only the extraction solvent was changed.

### 2.4. HPLC Analysis

CGA concentrations in the diluted extracts were determined using an E2695 high-performance liquid chromatography system (Waters, Milford, MA, USA) equipped with a photodiode array detector. Quantification was carried out with a CGA standard calibration curve covering 0.003125–0.100 mg/mL, which showed good linearity (r^2^ = 0.9994).

Separation was performed on an Atlantis T3 C18 column (4.6 mm × 250 mm, 5 μm; Waters) maintained at 30 °C. The detection wavelength was 328 nm. Mobile phase A was 0.2% (*w*/*v*) diammonium hydrogen phosphate aqueous solution, adjusted to pH 2.5 with concentrated phosphoric acid, and mobile phase B was acetonitrile. The analysis used isocratic elution with 85% A and 15% B at 1.0 mL/min. The injection volume was 60 μL, and all samples were filtered through a 0.22 μm PTFE syringe filter (13 mm; Tianjin Jinteng Experimental Equipment Co., Ltd., Tianjin, China) before injection.

### 2.5. Physicochemical Properties of Betaine-Acetic Acid DES

Betaine–acetic acid DES samples containing different proportions of water were prepared to evaluate their physicochemical properties. Viscosity was determined with an HR-20 rheometer (TA Instruments, New Castle, DE, USA) at temperatures ranging from 25 to 75 °C. Electrical conductivity was measured over the same temperature range using a DDSJ-319L conductivity meter (Leici Instrument Factory, Shanghai, China).

### 2.6. Single-Factor Experiments

After preliminary DES screening, betaine–acetic acid was selected as the extraction solvent for further optimization. Single-factor experiments were then conducted to evaluate the influence of five variables on CGA yield, namely the molar ratio of DES constituents, the water content of DES, liquid-to-solid ratio, extraction temperature, and extraction time. Unless otherwise specified, each experiment followed the extraction procedure described in Section 2.3, with only one parameter changed at a time while the other conditions were kept constant. The tested levels were as follows: molar ratio of DES constituents, 1:1–1:5; water content, 0–100% (*w*/*w*); liquid-to-solid ratio, 10–50 mL/g; extraction time, 20–60 min; and extraction temperature, 40–80 °C.

### 2.7. RSM Experimental Design

The single-factor results were used to define the variable ranges for response surface optimization. A Box–Behnken design (BBD) was constructed using three independent variables that strongly affected CGA extraction: liquid-to-solid ratio (A), extraction temperature (B), and extraction time (C). Each variable was set at three levels, and the BBD matrix is shown in Table 2. CGA yield (Y) was used as the response value, and the experimental data were fitted to a second-order polynomial model to determine the optimal extraction conditions.

### 2.8. FT-IR Spectroscopy

For FT-IR analysis, each dried sample was blended with potassium bromide (KBr), finely ground, and compressed into a transparent pellet. Spectra were collected on a Nicolet iS50 spectrometer (Thermo Fisher Scientific, Waltham, MA, USA) in the range of 4000–400 cm^−1^. Each spectrum was obtained with 32 scans at a spectral resolution of 4.0 cm^−1^.

### 2.9. SEM Observation

Solid residues obtained after DES and 70% ethanol extraction were separated by centrifugation at 8000 rpm for 10 min. After filtration to remove residual solvent, the residues were rinsed with ultrapure water. The recovered residues and untreated SCG powder were then dried at 60 °C until constant weight. Before SEM observation, all samples were sputter-coated with gold and examined using a Quanta 250 FEG field emission scanning electron microscope (FEI, Hillsboro, OR, USA) at an accelerating voltage of 20.00 kV.

### 2.10. UPLC-Q-TOF-MS Analysis for Crude Extract Profiling

The crude betaine–acetic acid DES extract obtained under the optimized UAE-DES conditions was filtered through a 0.22 μm PTFE syringe filter and then diluted 20-fold with 20% (*v*/*v*) methanol before instrumental analysis. A parallel extract prepared with 80% (*v*/*v*) methanol was analyzed as the reference sample. Chemical profiling was performed using an LC-30AD ultra-high-performance liquid chromatography system (Shimadzu, Kyoto, Japan) connected to a TripleTOF 5600+ high-resolution mass spectrometer (Sciex, Framingham, MA, USA). Analyst TF 1.6.1 software was used for data acquisition, and PeakView 2.1 software was used for data processing.

Separation was achieved on a Waters XBridge BEH C18 column (100 mm × 4.6 mm, 2.5 μm). The column temperature was maintained at 35 °C, with an injection volume of 5 μL and a flow rate of 0.5 mL/min. Mobile phase A consisted of water containing 0.1% (*v*/*v*) formic acid and 5 mmol/L ammonium formate, and mobile phase B was acetonitrile. The gradient program was as follows: 0–7 min, 95–10% A; 7–13 min, 10–0% A; 13–16 min, 0% A; 16–17 min, 0–95% A; and 17–20 min, 95% A for column re-equilibration.

Mass data were collected using an electrospray ionization (ESI) source in both positive (ESI+) and negative (ESI−) ion modes. Time-of-flight information-dependent acquisition mass spectrometry (TOF-IDA-MS) was applied. Automatic mass calibration was performed after every five samples using the manufacturer-provided calibration solution through a calibrant delivery system operated at 0.5 mL/min. Both the TOF-MS scan range and the IDA-MS/MS fragment ion range were set to 50–1500 Da. Spectra were acquired in high-sensitivity mode, and six spectra were recorded during each IDA cycle with dynamic background subtraction enabled.

The parameters for negative ESI mode were as follows: ion source temperature, 450 °C; curtain gas (N_2_), 35 psi; nebulizer gas (N_2_), 50 psi; heater gas (N_2_), 50 psi; ion spray voltage, −4500 V; declustering potential, −90 V; and collision energy, −15 to −55 eV. The corresponding positive ESI conditions were ion source temperature, 550 °C; curtain gas (N_2_), 30 psi; nebulizer gas (N_2_), 50 psi; heater gas (N_2_), 50 psi; ion spray voltage, +5000 V; declustering potential, +90 V; and collision energy, +15 to +55 eV.

Compound annotation was conducted by integrating accurate mass, retention behavior, isotope distribution, and MS/MS fragmentation information. Elemental compositions were proposed from high-resolution mass data, and possible structures were further checked against public databases and reported fragmentation patterns. When authentic standards were available, they were used to support compound confirmation. Compounds verified with authentic standards were regarded as confirmed, whereas those assigned from accurate mass and MS/MS evidence alone were described as tentatively identified. Representative phenolic acids and related metabolites are listed in the corresponding table in Section 3.6, and the complete annotation list is provided in Supplementary Table S1.

### 2.11. Isolation of CGA

For CGA isolation, the crude betaine–acetic acid DES extract was diluted 20-fold with 50% ethanol before loading. Before use, the macroporous resins were pretreated with hydrochloric acid and sodium hydroxide, and then washed with deionized water until neutral. Prior to column purification, five macroporous resins, including NKA-9, AB-8, ADS-21, D101, and X-5, were compared through preliminary static adsorption and desorption tests. ADS-21 was chosen for subsequent purification because it showed the best overall adsorption–desorption performance toward CGA.

Dynamic adsorption was then carried out using ADS-21 macroporous resin. The sample solution was loaded at a flow rate of 2 mL/min with a loading volume of 2 bed volumes (BV). Desorption was performed with 50% (*v*/*v*) ethanol at 2 BV. The eluted fraction was concentrated into powder using a rotary evaporator and stored at −20 °C until further analysis. The resin-screening and column-purification procedure was designed based on the preliminary adsorption/desorption tests and previous studies on CGA purification using macroporous resins or DES–resin integrated recovery systems, in which resin type, resin pretreatment, loading volume, flow rate, ethanol concentration, and elution volume were considered important parameters for CGA enrichment [23,24,25].

### 2.12. Antioxidant Activity Assays

#### 2.12.1. DPPH Radical Scavenging Assay

DPPH radical scavenging activity was tested according to a previously reported method with minor adjustments [26]. A 0.2 mM DPPH solution was prepared by dissolving 7.9 mg of DPPH in 100 mL of absolute ethanol. The purified coffee-residue CGA-rich extract (DES-E) was dissolved in 50% (*v*/*v*) ethanol and diluted to 6.25, 12.5, 25, 50, and 100 μg/mL. Vitamin C (VC), prepared in the same solvent and concentration range as the samples, was included as the positive control.

For each reaction, 1 mL of sample solution was combined with 1 mL of DPPH solution. The mixture was vortexed and kept at room temperature in the dark for 30 min. Absorbance was then recorded at 517 nm. The blank contained 1 mL of 50% ethanol and 1 mL of DPPH solution, while the sample background control contained 1 mL of sample solution and 1 mL of absolute ethanol. All measurements were performed three times.

The DPPH radical scavenging activity was calculated using Equation (1):

(1) $$\mathrm{DPPH}\;\mathrm{radical}\;\mathrm{scavenging}\;\mathrm{activity}\left(\%\right)=(1-\frac{A_{s}}{A_{c}})\times 100\%$$

where A_0_ is the absorbance of the blank, A_1_ is the absorbance of the sample–DPPH reaction mixture, and A_2_ is the absorbance of the sample background control. Results are expressed as mean ± standard deviation (SD).

#### 2.12.2. ABTS Radical Scavenging Assay

ABTS radical scavenging activity was determined according to a reported method [27]. The ABTS radical cation working solution was generated by mixing equal volumes of 2.5 mM potassium persulfate and 7 mM ABTS stock solution in PBS buffer. The mixture was allowed to react in the dark at room temperature for 12–16 h. Before analysis, the ABTS working solution was diluted with PBS buffer until the absorbance reached 0.70 ± 0.02 at 734 nm.

Sample solutions were prepared as described in Section 2.12.1. For the assay, 1 mL of sample solution was added to 4 mL of diluted ABTS working solution. After 30 min of dark incubation, absorbance was measured at 734 nm. Vitamin C was used as the positive control, and each measurement was repeated three times.

The ABTS radical scavenging activity was calculated using Equation (2):

(2) $$\mathrm{ABTS}\;\mathrm{radical}\;\mathrm{scavenging}\;\mathrm{activity}\left(\%\right)=(\frac{{A_{c}-A}_{s}}{A_{c}})\times 100\%$$

where Ac is the absorbance of the control mixture containing 1 mL of 50% ethanol and 4 mL of ABTS working solution, and As is the absorbance of the sample mixture containing 1 mL of sample solution and 4 mL of ABTS working solution. Results are expressed as mean ± SD.

#### 2.12.3. Ferric Reducing Antioxidant Power (FRAP) Assay

The reducing capacity of DES-E was evaluated using the FRAP assay. The FRAP working reagent was freshly prepared by mixing 10 volumes of 300 mM acetate buffer (pH 3.6), 1 volume of 10 mM TPTZ solution in 40 mM HCl, and 1 volume of 20 mM FeCl_3_·6H_2_O solution. The reagent was preheated to 37 °C before use. A calibration curve was established using FeSO_4_·7H_2_O standards at 0, 200, 400, 600, 800, 1000, 1200, and 1400 μM.

Because the FRAP and radical-scavenging assays rely on different reaction principles and absorbance responses, different sample concentration ranges were used. For FRAP analysis, DES-E was prepared at 40, 60, 80, 100, and 120 μg/mL in 50% (*v*/*v*) ethanol, whereas the DPPH and ABTS assays used 6.25–100 μg/mL. Vitamin C served as the positive control.

For each test, 0.2 mL of standard or sample solution was mixed with 2.8 mL of FRAP working reagent. The reaction was maintained at 37 °C in the dark for 30 min, followed by absorbance measurement at 593 nm. Each assay was conducted in triplicate. Reducing capacity was calculated from the FeSO_4_ standard curve and expressed as μM FeSO_4_ equivalents. Results are presented as mean ± SD.

### 2.13. Statistical Analysis

All measurements were carried out in triplicate, and the data are presented as mean ± standard deviation (SD). IBM SPSS Statistics 26 (IBM Corporation, Armonk, NY, USA), OriginPro 2021 (OriginLab Corporation, Northampton, MA, USA), and GraphPad Prism 9 (GraphPad Software, Boston, MA, USA) were used for statistical processing and figure preparation. Response surface modeling and optimization were performed using Design-Expert 13 (Stat-Ease, Minneapolis, MN, USA).

Student’s *t*-test was applied when two groups were compared. For datasets involving more than two groups, one-way analysis of variance (ANOVA) was used. Statistical significance was accepted at *p* < 0.05.

## 3. Results and Discussion

### 3.1. Screening of DESs for CGA Extraction

**Figure 1:**
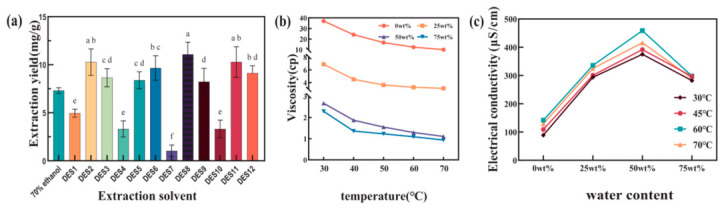
Screening and physicochemical properties of DESs: (**a**) effect of DES type on CGA extraction yield; (**b**) temperature-dependent conductivity of betaine–acetic acid DES with different water contents; and (**c**) temperature-dependent viscosity of betaine–acetic acid DES with different water contents. Significant difference analysis was performed using SPSS 26.0 software (*p* < 0.05). The different lowercase letters in the graph indicate significant differences at the 0.05 level.

Twelve DES systems prepared from choline chloride or betaine were compared for ultrasound-assisted CGA extraction from SCGs, with 70% ethanol included as the reference solvent. As shown in Figure 1a, CGA yield varied markedly among the 12 DESs tested, ranging from 5.23 to 11.07 mg CGA/g dried SCG powder. DES8, composed of betaine and acetic acid at a molar ratio of 1:2, gave the highest CGA yield of 11.07 mg CGA/g dried SCG powder, which was significantly higher than that obtained with 70% ethanol under the same preliminary extraction conditions (7.44 mg CGA/g dried SCG powder, *p* < 0.05).

The different extraction performances of the DESs can be explained by the combined effects of HBA and HBD structure on solvent polarity, hydrogen-bonding capacity, and matrix penetration. For betaine-based DESs, the zwitterionic structure of betaine provides both ionic and hydrogen-bonding interaction sites, which may favor the formation of a stable solvent network with suitable affinity for CGA [28]. By contrast, choline chloride-based DESs may show different hydration behavior and solvent–matrix interactions, leading to lower extraction efficiency under the tested conditions [29]. Among the HBDs examined, acetic acid appeared to provide a more suitable balance of acidity, polarity-related characteristics, and hydrogen-bonding ability for CGA dissolution.

The superior performance of DES8 may also be associated with the structural features of CGA. CGA contains hydroxyl, carboxyl, and ester groups, which can interact with DES components through hydrogen bonding and polarity-related interactions. Therefore, a DES system with an appropriate HBA–HBD combination can promote the release and solubilization of CGA from the SCG matrix. Based on these results, betaine–acetic acid DES was selected for subsequent single-factor experiments and response surface optimization.

### 3.2. Physical Properties of Betaine–Acetic Acid DES

To further understand the extraction behavior of the selected DES, the viscosity and electrical conductivity of betaine–acetic acid DES were measured at different temperatures and water contents. These two properties are closely associated with solvent mobility, ion migration, and mass transfer during extraction.

The betaine–acetic acid DES showed an initial viscosity of 4.98 mPa·s at room temperature, lower than that reported for several common DES systems such as choline chloride–acetic acid [30]. This relatively low viscosity is beneficial for extraction because it allows the solvent to penetrate the SCG matrix more easily and supports the diffusion of CGA into the liquid phase. As shown in Figure 1c, viscosity decreased continuously as temperature increased from 30 to 70 °C. This trend can be attributed to enhanced molecular motion and reduced internal friction at higher temperature. Increasing water content from 0 wt% to 75 wt% also reduced viscosity at each tested temperature, indicating that water weakened the hydrogen-bond-dominated supramolecular network of the DES and improved its fluidity [31].

Electrical conductivity was used to evaluate ion mobility in the DES system. As shown in Figure 1b, conductivity increased with water content at first and reached a maximum at 50 wt%, but it decreased when the water content was further increased to 75 wt%. The initial increase can be related to reduced viscosity and weakened intermolecular association, which favors ion migration. However, excessive water may dilute the DES microenvironment and shift the system toward an aqueous solution-like state, thereby reducing the effective concentration of mobile charge carriers [32,33].

Temperature also influenced the conductivity of the hydrated DES. Conductivity generally increased from 30 to 60 °C, consistent with enhanced ion mobility at higher temperature. However, a slight decrease was observed when the temperature rose from 60 to 70 °C, suggesting that the system did not follow a simple Arrhenius-type ion transport behavior. Such non-Arrhenius conductivity is often described by the Vogel–Fulcher–Tammann (VFT) equation, which is suitable for glassy, viscous, or strongly associated liquid systems where ion movement is coupled with free volume and structural relaxation [34]. In the present DES system, this deviation may reflect rearrangement of the hydrogen-bonding network, changes in free volume, or altered ion association at high temperature and high water content.

Taken together, the low viscosity of betaine–acetic acid DES provided favorable conditions for solvent penetration and mass transfer, while its conductivity behavior reflected the combined influence of temperature and water content on the DES microstructure. These physicochemical properties help explain why betaine–acetic acid DES showed high extraction efficiency for CGA from SCGs.

### 3.3. Single-Factor Experimental Results

Unless otherwise stated, the extraction temperature in the single-factor experiments was maintained using the temperature-control system of the ultrasonic bath.

#### 3.3.1. Effect of Molar Ratio of HBA to HBD on Extraction Yield

**Figure 2:**
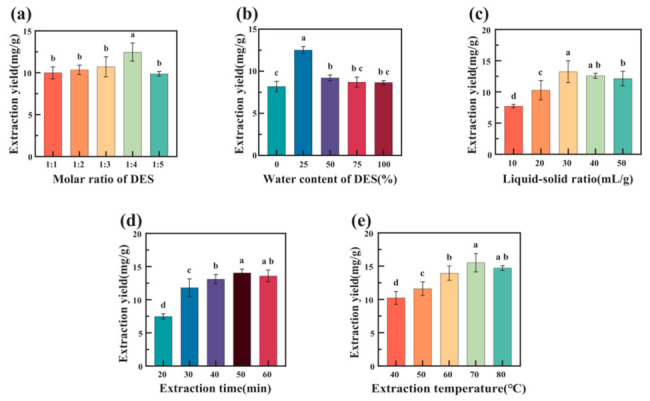
Effects of different variables on CGA yield: (**a**) molar ratio; (**b**) water content; (**c**) liquid-to-solid ratio; (**d**) extraction time; and (**e**) extraction temperature. Significant difference analysis was performed using SPSS 26.0 software (*p* < 0.05). The different lowercase letters in the graph indicate significant differences at the 0.05 level.

The influence of the betaine-to-acetic acid molar ratio was evaluated under the following fixed conditions: 25% water content, a liquid-to-solid ratio of 40 mL/g, an extraction temperature of 60 °C, and an extraction time of 40 min. As shown in Figure 2a, CGA yield increased as the proportion of acetic acid increased from 1:1 to 1:4 but declined when the ratio was further increased to 1:5. This result indicates that the HBA/HBD ratio strongly affected the extraction behavior of the DES system.

The improvement observed at moderate acetic acid levels may be associated with changes in the hydrogen-bonding network, viscosity, surface properties, and polarity-related characteristics of the solvent [35,36]. An appropriate amount of acetic acid can enhance solvent penetration into the SCG matrix and promote the dissolution and diffusion of CGA. However, excessive acetic acid may disturb the balance of intermolecular interactions within the DES and increase system acidity, which could weaken the extraction capacity. Therefore, a betaine-to-acetic acid molar ratio of 1:4 was used in the subsequent experiments.

#### 3.3.2. Effect of Water Content on Extraction Yield

Water content is an important factor regulating the fluidity and hydrogen-bonding structure of DESs. As shown in Figure 2b, CGA yield reached its maximum when the water content was 25%. At lower water content, the DES remained relatively viscous, which could limit solvent diffusion and reduce contact between the solvent and SCG particles. The addition of water lowered the viscosity and surface tension of the system, thereby improving matrix wetting, solvent penetration, and the effectiveness of ultrasound-assisted mass transfer [37].

When the water content exceeded 25%, the extraction yield decreased. Excessive water may over-dilute the DES microenvironment and disrupt the hydrogen-bond network responsible for interactions with CGA and related phenolic acids [38]. This weakening of the DES structure can reduce solvent affinity for the target compounds. Based on these results, 25% water content was selected for further optimization.

#### 3.3.3. Effect of Liquid-to-Solid Ratio on Extraction Yield

The effect of liquid-to-solid ratio was investigated from 10 to 50 mL/g while keeping the betaine-to-acetic acid molar ratio at 1:4, water content at 25%, extraction temperature at 60 °C, and extraction time at 40 min. As shown in Figure 2c, CGA yield increased with the liquid-to-solid ratio and reached 14.02 mg CGA/g dried SCG powder at 30 mL/g. A slight decrease was observed when the solvent volume was further increased.

A sufficient solvent volume is necessary to ensure effective contact with the SCG powder and to maintain a favorable concentration gradient for CGA diffusion. However, excessive solvent may disperse ultrasonic energy and reduce cavitation intensity per unit volume, which can weaken the physical disruption of the matrix. It also increases solvent consumption and downstream processing load. Considering both extraction performance and process economy, 30 mL/g was selected for the subsequent response surface design.

#### 3.3.4. Effect of Extraction Time on Extraction Yield

The effect of extraction time is shown in Figure 2d. CGA yield increased during the early stage of extraction and reached its maximum at 50 min. This increase can be explained by the gradual disruption of the SCG matrix and enhanced CGA release under ultrasonic cavitation. Prolonging the extraction time beyond 50 min did not further improve the yield and instead caused a slight decline.

The decrease at longer extraction time may be related to oxidative degradation of dissolved CGA or increased co-extraction of non-target substances, which could reduce the apparent extraction efficiency. Therefore, 50 min was chosen as the appropriate extraction time for subsequent optimization.

#### 3.3.5. Effect of Extraction Temperature on Extraction Yield

Extraction temperature also influenced CGA recovery. As shown in Figure 2e, CGA yield increased significantly as the temperature rose and reached a maximum at 70 °C. Higher temperature can lower DES viscosity, accelerate molecular movement, and improve mass transfer between the solvent and SCG matrix. These effects are favorable for CGA dissolution and diffusion.

When the temperature exceeded 70 °C, the extraction yield decreased. This reduction may be associated with thermal degradation of CGA and increased solvent loss at elevated temperature. Therefore, 70 °C was selected as the center point for the subsequent optimization experiment.

### 3.4. RSM Optimization of Extraction Conditions

Based on the single-factor results, liquid-to-solid ratio (A), extraction temperature (B), and extraction time (C) were selected as the variables for BBD optimization. CGA yield was taken as the response value. The experimental combinations and corresponding yields are shown in Table 2.

The relationship between the three variables and CGA yield was fitted using a quadratic regression model:

Y = 14.95 − 0.5050A + 1.15B − 0.1963C − 0.1100AB − 0.4750AC + 0.1675BC – 2.70A^2^ − 1.16B^2^ − 0.9038C^2^ (3)

where Y represents CGA yield (mg CGA/g dried SCG powder), A represents liquid-to-solid ratio (mL/g), B represents extraction temperature (°C), and C represents extraction time (min).

Table 3 summarizes the ANOVA results for the fitted model. The model showed a significant regression effect, as reflected by an F-value of 51.47 and *p* < 0.0001. The non-significant lack-of-fit result (*p* = 0.3190) indicated that the remaining deviation between predicted and experimental values was acceptable for the selected factor ranges. The values of R^2^ and R^2^adj were 0.9851 and 0.9660, respectively, confirming that the model described most of the experimental variation. The lower R^2^pred value of 0.8589 suggested that the model prediction was more sensitive to experimental variation than the model fitting. However, because R^2^pred remained above 0.8, the model was still considered adequate for predicting CGA yield within the investigated design space.

The relative influence of the three linear factors followed the order B > A > C, indicating that extraction temperature contributed most strongly to CGA yield. For the interaction terms, only AC was significant (*p* < 0.05), showing that liquid-to-solid ratio and extraction time jointly affected the extraction response. In contrast, AB and BC did not show significant interaction effects.

**Figure 3:**
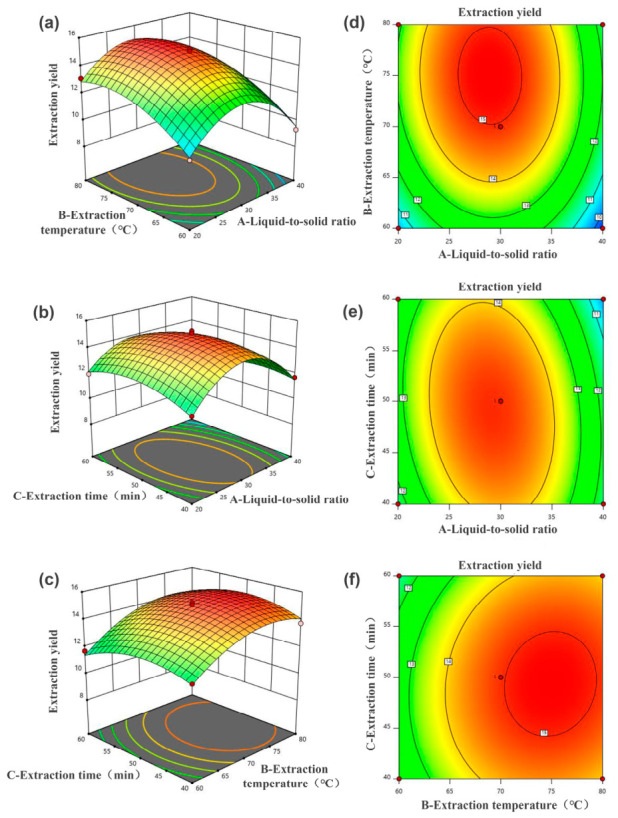
Response surface plots (**a**–**c**) and contour plots (**d**–**f**) showing the effects of liquid-to-solid ratio, extraction temperature, and extraction time on CGA yield.

The response surface plots supported the ANOVA results (Figure 3). The AC surface changed more sharply and showed a more elongated contour shape than the other interaction plots, which agreed with the significant AC term. The BC plot was comparatively flat, suggesting that the interaction between extraction temperature and extraction time was weak under the tested conditions. Thus, the graphical analysis and statistical results gave consistent interpretations.

The model predicted maximum CGA recovery at a liquid-to-solid ratio of 28.444 mL/g, extraction temperature of 75 °C, and extraction time of 49.5 min, with a predicted yield of 15.25 mg CGA/g dried SCG powder. For experimental validation, the conditions were rounded to 28 mL/g, 75 °C, and 50 min. The measured yield from three validation runs was 15.18 ± 0.23 mg CGA/g dried SCG powder, giving a relative error below 0.5%. This close agreement confirmed that the optimized model could guide CGA extraction using the betaine–acetic acid DES system.

These optimized parameters were specific to the betaine–acetic acid DES system. Therefore, the extraction experiment using 70% ethanol was included only as a parallel reference under the DES-optimized conditions rather than as an independently optimized ethanol process. Under this comparison framework, 70% ethanol produced a CGA yield of 7.89 ± 0.31 mg CGA/g dried SCG powder, confirming the higher extraction performance of the DES system.

### 3.5. Experimental Evidence for the DES-Assisted Extraction Mechanism

FT-IR and SEM were used to support the proposed extraction mechanism from two experimental perspectives: intermolecular interactions in the DES and structural changes in the SCG matrix. Because no molecular dynamics simulation, density functional theory calculation, or quantitative intermolecular interaction analysis was conducted, the following discussion is limited to spectroscopic and microscopic evidence.

#### 3.5.1. FT-IR Evidence for Intermolecular Interactions in the DES

**Figure 4:**
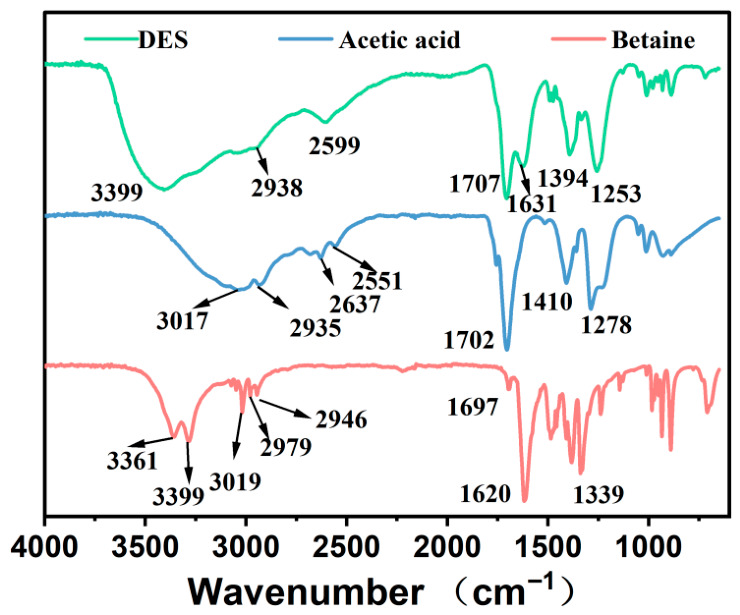
FT-IR spectra of betaine, acetic acid, and the betaine–acetic acid DES.

FT-IR spectra were collected for betaine, acetic acid, and the corresponding betaine–acetic acid DES to examine whether new intermolecular interactions were formed after DES preparation. As shown in Figure 4, the spectrum of the DES differed from those of the two individual components, suggesting that the local chemical environments of the functional groups were changed after mixing.

For acetic acid, the broad O–H stretching region and the C=O stretching band at 1702 cm^−1^ were associated with its carboxyl group. Betaine showed characteristic bands related to carbonyl/carboxylate vibration and C–N vibration at 1697 and 1339 cm^−1^, together with C–H stretching signals at 2946 and 3019 cm^−1^. In the DES spectrum, the O–H-related band became broader and appeared at 3399 cm^−1^, while the carbonyl/carboxylate-related bands shifted to 1707 and 1410 cm^−1^. These changes indicate that betaine and acetic acid interacted through hydrogen bonding and formed a new supramolecular solvent network.

This hydrogen-bond-rich environment may contribute to CGA extraction because CGA and related phenolic acids contain hydroxyl, ester, and carboxyl groups that can interact with DES components. Such multipoint interactions can improve the affinity of the solvent toward phenolic acids, thereby supporting the selective enrichment observed in the UPLC-Q-TOF-MS analysis.

#### 3.5.2. Morphological Changes in SCGs After Extraction

**Figure 5:**
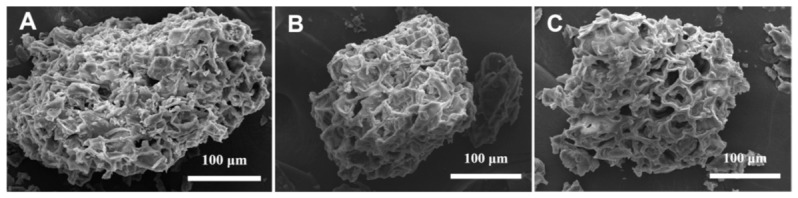
SEM images of SCGs: (**A**) untreated sample; (**B**) 70% ethanol-treated sample; and (**C**) betaine–acetic acid DES-treated sample.

SEM observation was used to compare the surface morphology of untreated SCGs, 70% ethanol-treated SCGs, and betaine–acetic acid DES-treated SCGs. As shown in Figure 5A–C, the untreated sample retained a relatively compact surface, indicating that the original SCG matrix was structurally intact before extraction. After treatment with 70% ethanol, the particles showed surface wrinkling and partial damage, suggesting that ethanol extraction produced only moderate disruption of the matrix.

In contrast, the DES-treated sample displayed more evident surface rupture, irregular fragments, and a rougher porous structure. This morphological change suggests that the betaine–acetic acid DES, combined with ultrasound, promoted stronger matrix disruption than ethanol under the tested conditions. Ultrasound-induced cavitation and shear effects may weaken the rigid cell-wall structure, whereas the low viscosity and hydrogen-bonding capacity of the DES can facilitate solvent infiltration into the matrix. The increased solvent–solid contact area then favors the release and diffusion of CGA and related phenolic acids [39].

Overall, the FT-IR and SEM results provide complementary experimental evidence for the extraction mechanism. The DES promoted phenolic acid solubilization through hydrogen-bonding interactions, while ultrasound-assisted structural disruption improved mass transfer from the SCG matrix to the solvent phase.

### 3.6. UPLC-Q-TOF-MS Analysis of the Chemical Profile of the DES Extract

UPLC-Q-TOF-MS was used to characterize the chemical composition of the optimized betaine–acetic acid DES extract in both positive and negative ion modes. Compound annotation was performed by considering accurate mass, retention behavior, and MS/MS fragmentation patterns. Table 4 lists representative compounds related to the main chemical features of the DES extract, while the complete annotation results are provided in Supplementary Table S1.

**Figure 6:**
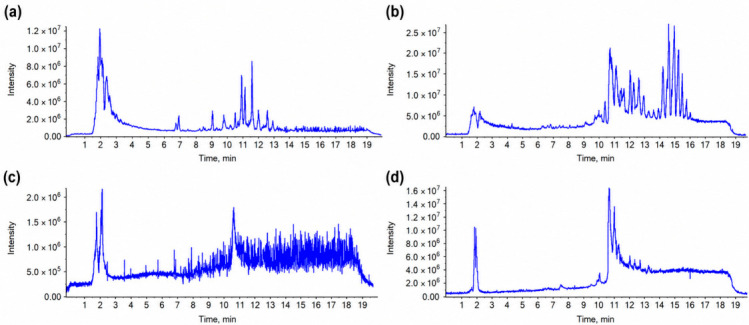
Total ion chromatograms: (**a**) 80% methanol extract in negative ion mode; (**b**) 80% methanol extract in positive ion mode; (**c**) betaine–acetic acid DES extract in negative ion mode; and (**d**) betaine–acetic acid DES extract in positive ion mode.

The optimized betaine–acetic acid DES extract showed a chemical profile dominated by phenolic acids and chlorogenic-acid-related derivatives. The representative compounds included CGA, caffeic acid, ferulic acid, quinic acid, 3-O-feruloylquinic acid, 3,4-dicaffeoylquinic acid, and 3-caffeoylquinic acid lactone. Naringenin was also detected as a minor flavonoid component. This profile indicates that the betaine–acetic acid DES extract was mainly enriched in phenolic-acid-type constituents while still retaining a certain degree of chemical diversity, consistent with the total ion chromatograms shown in Figure 6.

A semi-quantitative comparison with the 80% methanol extract further reflected the selectivity of the betaine–acetic acid DES system. Although the betaine–acetic acid DES extract was diluted 20-fold before injection, several phenolic markers, including CGA, quinic acid, ferulic acid, and 3-O-feruloylquinic acid, still produced clear MS signals. In contrast, caffeine and lipophilic fatty acids, such as linoleic acid and palmitic acid, were less prominent under the same analytical conditions. This pattern suggests that the DES system preferentially extracted hydroxyl- and carboxyl-containing phenolic compounds rather than less polar co-extractives.

The compositional characteristics observed by UPLC-Q-TOF-MS were consistent with the FT-IR and SEM evidence discussed above. The hydrogen-bonding network formed by betaine and acetic acid may increase the affinity of the solvent for phenolic acids, whereas ultrasound-assisted disruption of the SCG matrix can facilitate the release of these compounds into the solvent phase. Therefore, the UPLC-Q-TOF-MS results provide chemical evidence that the optimized UAE-DES process generated a phenolic-acid-rich extract from SCGs.

The detected phenolic constituents also provide a chemical basis for the antioxidant activity evaluated in the following section. CGA, caffeic acid, ferulic acid, and hydroxycinnamic acid derivatives contain phenolic hydroxyl groups and conjugated structures that can contribute to radical scavenging and reducing capacity. Thus, the UPLC-Q-TOF-MS profile supports the subsequent interpretation that the antioxidant activity of the purified extract is closely associated with the phenolic-acid-rich composition obtained through UAE-DES extraction.

### 3.7. Evaluation of Antioxidant Activity

**Figure 7:**
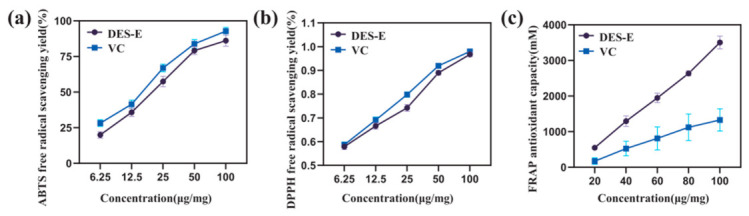
Antioxidant activity of the purified coffee-residue CGA-rich extract (DES-E): (**a**) ABTS radical scavenging activity; (**b**) DPPH radical scavenging activity; and (**c**) ferric reducing antioxidant power (FRAP).

The antioxidant properties of the purified coffee-residue CGA-rich extract obtained after macroporous resin treatment (DES-E) were examined using ABTS, DPPH, and FRAP assays, with vitamin C (VC) as the positive control. As shown in Figure 7, DES-E produced clear antioxidant responses in all three assays, although its activity relative to VC differed among the assay systems.

For ABTS radical scavenging (Figure 7a), the activity of DES-E increased with increasing sample concentration. At 100 μg/mL, DES-E reached a scavenging rate of 86.14 ± 1.75%, approaching the value recorded for VC at the same concentration (92.76 ± 1.21%). In the DPPH assay (Figure 7b), DES-E also showed a strong scavenging response, with an inhibition rate of 93.36 ± 0.46% at 100 μg/mL. In the FRAP assay (Figure 7c), DES-E showed a reducing capacity of 3506 μM FeSO_4_ equivalents at 100 μg/mL, exceeding that of VC under the tested conditions. These results indicate that DES-E exhibited strong antioxidant activity, but its relative performance should be discussed according to the specific assay rather than described as uniformly superior to VC.

The antioxidant behavior of DES-E is closely related to its phenolic-acid-rich composition. UPLC-Q-TOF-MS analysis of the betaine–acetic acid DES extract before purification confirmed the presence of CGA and other phenolic constituents, including caffeic acid, ferulic acid, quinic acid derivatives, and hydroxycinnamic acid derivatives [40]. After purification with macroporous resin, these phenolic-acid-related compounds were concentrated in DES-E [41,42]. Their phenolic hydroxyl groups and conjugated structures can participate in hydrogen donation, radical scavenging, and electron-transfer reactions, which explains the ABTS, DPPH, and FRAP responses observed in this study.

The antioxidant results are therefore consistent with the selective enrichment of phenolic-acid-related constituents by the betaine–acetic acid DES system. These findings further support the potential of UAE-DES for preparing CGA-rich antioxidant extracts from SCGs.

## 4. Conclusions

This study confirmed that ultrasound-assisted extraction with betaine–acetic acid DES is an effective approach for recovering CGA and related phenolic acids from SCGs. Under the optimized DES conditions, the CGA yield reached 15.18 ± 0.23 mg CGA/g dried SCG powder, while 70% ethanol used as a parallel reference under the same DES-optimized conditions gave a lower yield.

The improved extraction behavior was supported by structural and compositional analyses. FT-IR results indicated that betaine and acetic acid formed a hydrogen-bonding network, which may favor interactions with phenolic acids. SEM images showed greater disruption of the SCG matrix after UAE-DES treatment than after ethanol extraction, suggesting enhanced solvent penetration and mass transfer. UPLC-Q-TOF-MS analysis further confirmed that the optimized DES extract contained abundant phenolic-acid-related constituents, including CGA, caffeic acid, ferulic acid, and hydroxycinnamic acid derivatives.

After purification with macroporous resin, the purified coffee-residue CGA-rich extract (DES-E) displayed strong antioxidant activity in ABTS, DPPH, and FRAP assays. Its activity compared with VC differed among the three assays, indicating that the antioxidant performance of DES-E should be interpreted according to the specific reaction system. The observed activity was closely associated with the enrichment of CGA and related phenolic acids.

Overall, the UAE-DES process based on betaine–acetic acid provides a green and effective route for producing CGA-rich antioxidant extracts from SCGs. Future studies should further examine DES recycling, extract safety, bioavailability, and potential application in food or nutraceutical products.

## Figures and Tables

**Table 1:** Composition and polarity-related information of the deep eutectic solvents used for CGA extraction.

Name	HBA	HBD	Molar Ratio (HBA:HBD)	Water Content (% *w*/*w*)	Polarity-Related Solvent Type
DES1	Choline chloride	Sorbitol	1:2	25	Polyol-based polar DES
DES2	Choline chloride	Acetic acid	1:2	25	Organic-acid-based polar DES
DES3	Choline chloride	1,4-Butanediol	1:2	25	Diol-based DES
DES4	Choline chloride	Citric acid	1:2	25	Organic-acid-based highly polar DES
DES5	Choline chloride	Ethylene glycol	1:2	25	Diol-based polar DES
DES6	Choline chloride	Propylene glycol	1:2	25	Diol-based polar DES
DES7	Betaine	Sorbitol	1:2	25	Polyol-based polar DES
DES8	Betaine	Acetic acid	1:2	25	Organic-acid-based polar DES
DES9	Betaine	1,4-Butanediol	1:2	25	Diol-based DES
DES10	Betaine	Citric acid	1:2	25	Organic-acid-based highly polar DES
DES11	Betaine	Ethylene glycol	1:2	25	Diol-based polar DES
DES12	Betaine	Propylene glycol	1:2	25	Diol-based polar DES

Note: All DESs contained 25% (*w*/*w*) water. Because quantitative polarity parameters were not determined in this study, the polarity-related solvent types were assigned according to the HBD category and the expected hydrogen-bonding or polar characteristics of the DES components.

**Table 2:** Experimental Design and Results of the BBD.

Run	A: Liquid-to-Solid Ratio (mL/g)	B: Extraction Temperature (°C)	C: Extraction Time (min)	Extraction Yield (mg CGA/g Dried SCG Powder)
1	20	60	50	10.18
2	40	60	50	9.30
3	20	80	50	13.10
4	40	80	50	11.78
5	20	70	40	11.66
6	40	70	40	11.69
7	20	70	60	11.96
8	40	70	60	10.09
9	30	60	40	12.17
10	30	80	40	13.73
11	30	60	60	11.70
12	30	80	60	13.93
13	30	70	50	14.49
14	30	70	50	14.78
15	30	70	50	15.18
16	30	70	50	15.24
17	30	70	50	15.06

**Table 3:** ANOVA for the regression model.

Source	Sum of Squares	df	Mean Square	F-Value	*p*-Value	Significance
Model	57.07	9	6.340	51.47	<0.0001	**
A	2.040	1	2.040	16.56	0.0048	**
B	10.560	1	10.560	85.69	<0.0001	**
C	0.308	1	0.308	2.50	0.1578	ns
AB	0.048	1	0.048	0.39	0.5507	ns
AC	0.902	1	0.902	7.33	0.0304	*
BC	0.112	1	0.112	0.91	0.3717	ns
A^2^	30.610	1	30.610	248.45	<0.0001	**
B^2^	5.700	1	5.700	46.28	0.0003	**
C^2^	3.440	1	3.440	27.91	0.0011	**
Residual	0.862	7	0.123			
Lack of fit	0.472	3	0.157	1.62	0.319	ns
Pure Error	0.389	4	0.097			
Cor Total	57.930	16				

Note: A, liquid-to-solid ratio; B, extraction temperature; C, extraction time. ** *p* < 0.01; * *p* < 0.05; ns, not significant.

**Table 4:** Representative Phenolic Acids and Related Metabolites Identified in the Betaine–Acetic Acid DES Extract by UPLC-Q-TOF-MS.

No.	t_R_ (min)	Compound Name	Formula	Measured (*m*/*z*)	Structural Assignment
1	1.7807	Chlorogenic acid	C_16_H_18_O_9_	353.08769	Hydroxycinnamoylquinic acid
2	2.2799	Caffeic acid	C_9_H_8_O_4_	179.03449	Hydroxycinnamic acid
3	2.3525	Ferulic acid	C_10_H_10_O_4_	193.04990	Hydroxycinnamic acid
4	1.7652	Quinic acid	C_7_H_12_O_6_	191.05597	Quinic acid/CGA-related backbone
5	2.3687	Vanillic acid	C_8_H_8_O_4_	167.03464	Hydroxybenzoic acid
6	1.8004	3-O-Feruloylquinic acid	C_17_H_20_O_9_	367.09995	Acylquinic acid derivative
7	1.7906	3,4-Dicaffeoylquinic acid	C_25_H_24_O_12_	515.11908	Dicaffeoylquinic acid derivative
8	2.0391	3-Caffeoylquinic acid lactone	C_16_H_16_O_8_	335.07654	Chlorogenic-acid-related lactone
9	2.2482	Naringenin	C_15_H_12_O_5_	271.05947	Flavonoid
10	2.2126	Caffeine	C_8_H_10_N_4_O_2_	195.08753	Alkaloid
11	9.5812	Palmitic acid	C_16_H_32_O_2_	255.23175	Fatty acid
12	8.8294	Linoleic acid	C_18_H_32_O_2_	279.23305	Fatty acid

Note: Representative compounds were selected from the full annotation list to show the major phenolic-acid-related features of the optimized betaine–acetic acid DES extract. Compound assignments were tentatively made according to accurate mass, retention behavior, and MS/MS fragmentation information obtained from UPLC-Q-TOF-MS analysis. The full annotation list is provided in Supplementary Table S1. The DES extract was diluted 20-fold with 20% methanol before injection.

## Data Availability

The original contributions presented in this study are included in the article/Supplementary Material. Further inquiries can be directed to the corresponding author.

## Supplementary Materials

The following supporting information can be downloaded at: https://www.mdpi.com/article/10.3390/foods15101743/s1, Table S1, Tentatively identified compounds in the 80% methanol extract and betaine–acetic acid DES extract by UPLC-Q-TOF-MS.

## Abbreviations

ABTS, 2,2′-azino-bis(3-ethylbenzothiazoline-6-sulfonic acid); BBD, Box–Behnken design; BV, bed volume; CGA, chlorogenic acid; DES, deep eutectic solvent; DES-E, purified coffee-residue CGA-rich extract; DPPH, 1,1-diphenyl-2-picrylhydrazyl; ESI, electrospray ionization; FT-IR, Fourier-transform infrared spectroscopy; FRAP, ferric reducing antioxidant power; HBA, hydrogen bond acceptor; HBD, hydrogen bond donor; HPLC, high-performance liquid chromatography; IDA, information-dependent acquisition; RSM, response surface methodology; SCGs, spent coffee grounds; SEM, scanning electron microscopy; TPTZ, 2,4,6-tris(2-pyridyl)-s-triazine; UAE, ultrasound-assisted extraction; UPLC-Q-TOF-MS, ultra-performance liquid chromatography–quadrupole time-of-flight mass spectrometry; VC, vitamin C.
