# Supplementary material for: Selective Enrichment of Chlorogenic Acid and Related Phenolic Acids from Spent Coffee Grounds by Ultrasound-Assisted Extraction with Deep Eutectic Solvents
Source: Foods. 2026 May 14;15(10):1743. doi: 10.3390/foods15101743 (PMC13205755; doi:10.3390/foods15101743)
Supplement: Supplementary file 1 [file foods-15-01743-s001.zip › foods-4259228-supplementary.pdf]

Table S1. Tentatively identified compounds in the 80% methanol extract and betaine–acetic acid DES extract by UPLC-Q-TOF-MS

Table S1A. Compounds identified in ESI $^+$ mode

**No.**	**Compound name**	**Category**	**Theoretical** *m/z*	**Measured** *m/z*	**Methanol peak area**	**DES peak area**
1	Caffeine	Alkaloid	195.08765	195.08753	106456	7074
2	Trigonelline	Alkaloid	138.05496	138.05546	27117	232
3	Choline	Choline derivative	104.10754	104.10785	2369	40
4	Glycerophosphoch oline	Choline derivative	258.1101	258.11041	1006	15
5	O-Acetylcholine	Choline derivative	146.1181	146.11809	284	0
6	Rutin	Flavonoid	611.16066	611.16102	129	104

Table S1B. Compounds identified in ESI ⁻ mode

No.	Compound name	Category	Theoretical *m/z*	Measured *m/z*	Methanol peak area	DES peak area
1	Chlorogenic acid	Phenolic acid	353.08781	353.08769	58168	6740
2	Caffeic acid	Phenolic acid	179.03498	179.03449	4443	379
3	Ferulic acid	Phenolic acid	193.05063	193.0499	10871	1572
4	Cafestol	Diterpene / other	315.19657	315.19669	151	20
5	5-Hydroxymethylfurfural (5-HMF)	Other	125.02442	125.02444	678	27
6	Palmitic acid	Fatty acid	255.23295	255.23175	45100	1353
7	Linoleic acid	Fatty acid	279.23295	279.23305	75414	30
8	Malic acid	Organic acid	133.01425	133.01395	3647	699
9	Citric acid	Organic acid	191.01973	191.01883	27816	1327
10	Gluconic acid	Organic acid	195.05103	195.05151	629	14
11	2-Hydroxyglutaric acid	Organic acid	147.0299	147.02966	1364	205
12	Quinic acid	Organic acid / CGA-related	191.05611	191.05597	83330	12464
13	Protocatechuic acid	Phenolic acid	153.01933	153.01913	523	19
14	Protocatechualdehyde	Phenolic aldehyde	137.02442	137.02348	6647	396
15	p-Coumaric acid	Phenolic acid	163.04007	163.03871	1436	164
16	Vanillic acid	Phenolic acid	167.03498	167.03464	1272	21
17	Gallic acid	Phenolic acid	169.01425	169.01316	65	6
18	3,4-Dimethoxycinnamic acid	Phenolic acid derivative	207.06628	207.06673	2015	232
19	trans-Caffeoyl-L-tryptophan sodium salt	Phenolic acid-related derivative	365.1143	365.1159	1063	126
20	Coumaroyltryptophan	Phenolic acid-related derivative	349.11938	349.11925	628	16
21	3-Caffeoylquinic acid lactone	CGA-related derivative	335.07724	335.07654	118404	1609
22	3,4,5-Tricaffeoylquinic acid	CGA-related derivative	677.15119	677.1501	822	12
23	3-O-Feruloylquinic acid	CGA-related derivative	367.10346	367.09995	12544	1235
24	3,4-Dicaffeoylquinic acid lactone	CGA-related derivative	497.10894	497.10895	3074	90
25	3,4-Dicaffeoylquinic acid	CGA-related derivative	515.1195	515.11908	9241	1038
26	3-Caffeoyl-5-feruloylquinic acid	CGA-related derivative	529.13515	529.13543	1410	12
27	4-Caffeoyl-5-feruloylquinic acid	CGA-related derivative	529.13515	529.13644	1309	142
28	Atractyligenin	Terpenoid / other	319.19148	319.18999	2410	210
29	Phenylalanine	Amino acid	164.0717	164.07095	628	10
30	Naringenin	Flavonoid	271.0612	271.05947	5376	240
31	Naringenin glucoside	Flavonoid glycoside	433.11402	433.11158	6428	145
32	Quercetin glucoside	Flavonoid glycoside	463.0882	463.08832	492	18
33	Pantothenic acid	Organic acid / vitamin	218.1034	218.1028	273	15
34	Sucrose	Sugar	341.10894	341.10866	3089	77
35	Quercetin	Flavonoid	301.03538	301.03729	45	0
36	Kaempferol	Flavonoid	285.04046	285.03908	69	0

Table note:

A total of 42 compounds were tentatively identified under positive- and negative-ion modes, including 6 compounds in ESI^+ mode and 36 compounds in ESI^- mode. The DES extract was diluted 20-fold with 20% methanol prior to injection, and the 80% methanol extract was analyzed as the control. Peak areas therefore reflect relative MS responses under the same analytical conditions.
